# Functional characterization of NES and GES responsible for the biosynthesis of (*E*)-nerolidol and (*E,E*)-geranyllinalool in *Tripterygium wilfordii*

**DOI:** 10.1038/srep40851

**Published:** 2017-01-27

**Authors:** Ping Su, Tianyuan Hu, Yujia Liu, Yuru Tong, Hongyu Guan, Yifeng Zhang, Jiawei Zhou, Luqi Huang, Wei Gao

**Affiliations:** 1 School of Traditional Chinese Medicine, Capital Medical University, Beijing, China; 2 State Key Laboratory Breeding Base of Dao-di Herbs, National Resource Center for Chinese Materia Medica, China Academy of Chinese Medical Sciences, Beijing, China; 3 Beijing Key Lab of TCM Collateral Disease Theory Research, Beijing, China

## Abstract

Triptolide and celastrol, two principal bioactive compounds in *Tripterygium wilfordii*, are produced from geranylgeranyl diphosphate (GGPP) and farnesyl diphosphate ((*E,E*)-FPP) through terpenoid biosynthesis pathway. However, little is known about *T. wilfordii* terpene synthases which could competitively utilize GGPP and (*E,E*)-FPP as substrates, producing C_15_ and C_20_ tertiary alcohols. Here we firstly cloned the genes encoding nerolidol synthase (NES) and geranyllinalool synthases (GES1, GES2), which are responsible for the biosynthesis of (*E*)-nerolidol and (*E,E*)-geranyllinalool. *In vitro* characterization of recombinant TwNES and TwGES1 revealed both were functional enzymes that could catalyze the conversion of (*E,E*)-FPP and GGPP to (*E*)-nerolidol and (*E,E*)-geranyllinalool, which were consistent with the results of yeast fermentation. Biochemical characterization revealed TwNES and TwGES1 had strong dependency for Mg^2+^, *K*_m_ and *K*_cat_/*K*_m_ values of TwNES for (*E,E*)-FPP were 12.700 μM and 0.029 s^−1^/μM, and TwGES1 for GGPP were 2.039 μM and 0.019 s^−1^/μM. Real-time PCR analysis showed the expression levels of *NES* and *GES1* increased by several fold in the suspension cells treated with alamethicin, indicating TwNES and TwGES1 are likely to utilize GGPP and (*E,E*)-FPP to generate tertiary alcohols as precursor of plant volatiles, which play important roles in the ecological interactions between *T. wilfordii* and other organisms.

*Tripterygium wilfordii* Hook. f., also known as lei gong teng, is widely used in the treatment of rheumatoid arthritis and other inflammatory diseases in Traditional Chinese Medicine^1^,^2^. The main effective compounds in the plants are terpenoids, whose precursors include diterpenes derived from geranylgeranyl diphosphate (GGPP), triterpenes from farnesyl diphosphate ((*E,E*)-FPP) and monoterpenes from geranyl diphosphate (GPP)^3^,^4^,^5^,^6^. Among these terpenoids, the sesquiterpene alcohol (*E*)-nerolidol and diterpene alcohol (*E,E*)-geranyllinalool, confirmed as the precursors of plant defense compounds, are widely distributed in the plant kingdom^7^,^8^,^9^,^10^,^11^ and competitively utilize GGPP, (*E,E*)-FPP or GPP as substrates with other terpenoids.

The biosynthesis pathway leading to (*E*)-nerolidol and (*E,E*)-geranyllinalool, same as that of other terpenoids in higher plants, starts from the condensation of isopentenyl diphosphate (IPP) and dimethylallyl diphosphate (DMAPP), which formed through either the cytoplasmic mevalonate (MVA) or the plastidic 2-C-methyl-D-erythritol-4-phosphate (MEP) pathway^12^. Sequential condensations of these two C_5_-units, result in the formation of linear elongated prenyldiphosphates, including GPP, (*E,E*)-FPP and GGPP^13^,^14^,^15^. Next, nerolidol synthase (NES) or geranyllinalool synthase (GES) catalyzes a two-step reaction, in which carbocation formation of the (*E,E*)-FPP or GGPP is followed by an allylic rearrangement, resulting in the production of the tertiary alcohol (*E*)-nerolidol or (*E,E*)-geranyllinalool^7^,^16^ (Fig. 1).

To date, scores of enzymes involved in terpenoid biosynthesis pathway, responsible for the formation of the bioactive compounds (e.g. triptolide and celastrol), have been identified^3^,^5^,^6^,^17^,^18^,^19^. None of enzymes, impacting the contents of triptolide and celastrol by consuming their precursor compounds competitively or affecting the plant growth through regulating the production of defense compounds, have been reported. In this manuscript, we firstly clone the full-length genes encoding NES and GES enzymes in *T. wilfordii* suspension cells and identify the functions of the enzymes both *in vitro* and in yeast. Both of *TwNES* and *TwGES1* transcript levels are upregulated simultaneously in suspension cells by treatment with alamethicin, suggesting that TwNES and TwGES1 may participated in the ecological interactions between *T. wilfordii* and other organisms through regulating the synthesis of plant defense volatiles. Functional characterization of TwNES and TwGES provide two gene regulatory elements for further regulating the biosynthesis of the bioactive compounds (e.g. triptolide and celastrol) in *T. wilfordii*.

## Results

### Cloning and sequence analysis of *TwNES* and *TwGES*

Previous reports indicated that nerolidol synthase (NES) and geranyllinalool synthase (GES) utilize GGPP, (*E,E*)-FPP or GPP as substrates^15^,^16^, which were two competitors of the triptolide and celastrol biosynthetic pathway in *T. wilfordii*. Thus, the identification of them in *T. wilfordii* would facilitate the understanding of the synthesis route of triptolide and celastrol. From the *T. wilfordii* transcriptome sequencing dataset, the *TwNES, TwGES* gene sequences were screened out and cloned using the cDNA of suspension cells.

The full length *TwNES* cDNA (GenBank accession number KU588405) is 1891 nt and encodes a polypeptide of 552 amino acids. The compute isoelectric point (PI) and molecular weight (MW) of TwNES are 5.33/63.02 kDa. TwNES was homologous to NES in other species according to the multiple alignments (Supplementary Figure 1). The protein domain analysis showed that TwNES has a terpenoid cyclases/protein prenyltransferase alpha-alpha toroid between 35–227 aa and a TPS pfam between 62–202 aa. The TPS metal-binding domain was between 232–551 aa.

The *TwGES1* cDNA (GenBank accession number KU577438) is 2946 nt in length and encodes a predicted protein of 848 amino acid residues (PI: 5.76, MW: 97.89 kDa). The *TwGES2* cDNA (GenBank accession number KU577439) is 2700 nt in length and has an open reading frame (ORF) encoding 766 amino acid residues (PI: 5.84, MW: 88.35 kDa). The sequences of the two *TwGES*s are basically the same (similarity identified as 90.45%) except that *TwGES2* misses a fragment (from the 1825^th^ to the 2070^th^ nt) versus *TwGES1*, leading the TwGES2 protein misses 82 amino acids. TwGES1 was homologous to GES in other species according to the multiple alignments (Supplementary Figure 2). The protein domain analysis showed that TwGES1 has a terpenoid cyclases/protein prenyltransferase alpha-alpha toroid between 33–152 aa and a TPS pfam between 231–404 aa. The TPS metal-binding domain was between 446–766 aa. The “missing amino acids” of TwGES2 versus TwGES1 was in the TPS metal binding domain.

### Phylogenetic Tree Construction

**Figure 2:**
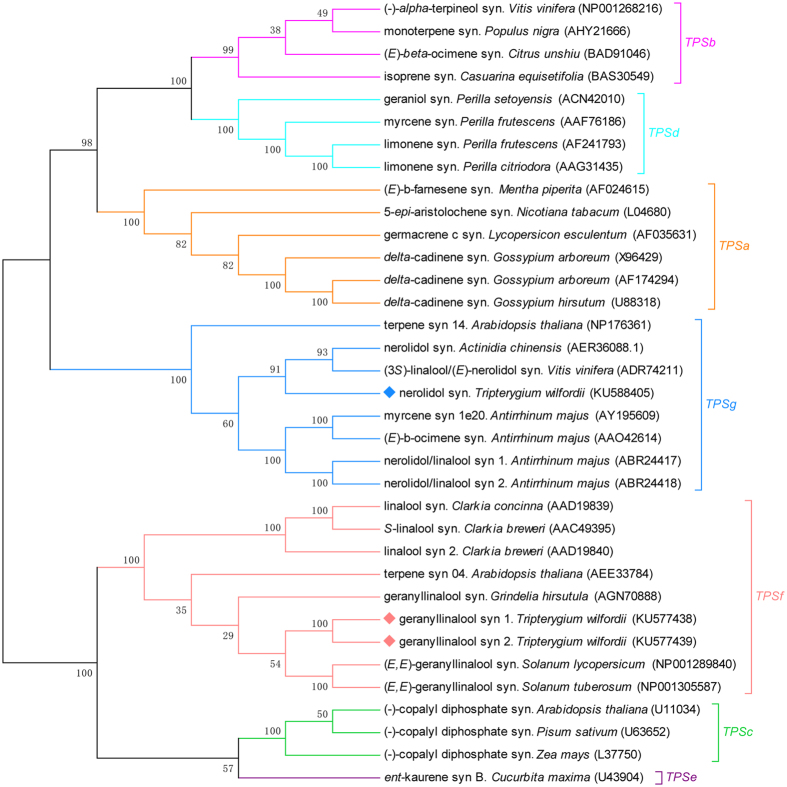
Phylogenetic analysis of selected terpene synthases from different species. TwNES, TwGES1 and TwGES2 are indicated by filled square. The Neighbor-Joining phylogenetic trees were constructed using the bootstrap method on MEGA 5.1 and the number of Bootstrap replications was 1000.

The phylogenetic tree was constructed based on the TPS from other species downloaded from NCBI database (Fig. 2). It is obvious that NESs from different plants clustered while the GESs in different species clustered in the other clade. TwNES clustered with the TPS enzymes in the TPS-g subgroup. The TPS-g enzymes lack an N-terminal RRX_8_W motif, which is present in many terpene synthases and also reported to be required to the catalytic functions of the monoterpene synthases in the angiosperm TPS-b and gymnosperm TPS-d enzyme clade^20^,^21^,^22^,^23^. TwNES clustered most closely to the (3 *S*)-linalool/(*E*)-nerolidol synthase from *Vitis vinifera*^24^, with 61% amino acid identity. TwGES1 and TwGES2 clustered with TPS enzymes in the TPS-f clade, but showed a farther genetic distance from GESs of other plants. TwGES1 and TwGES2 clustered most closely to the terpene synthase from *Solanum lycopersicum*^25^, with 47% amino acid identity, indicating a similar enzyme catalysis that they may have.

### Functional characterization of TwNES and TwGES both *in vitro* and in yeast

**Figure 3:**
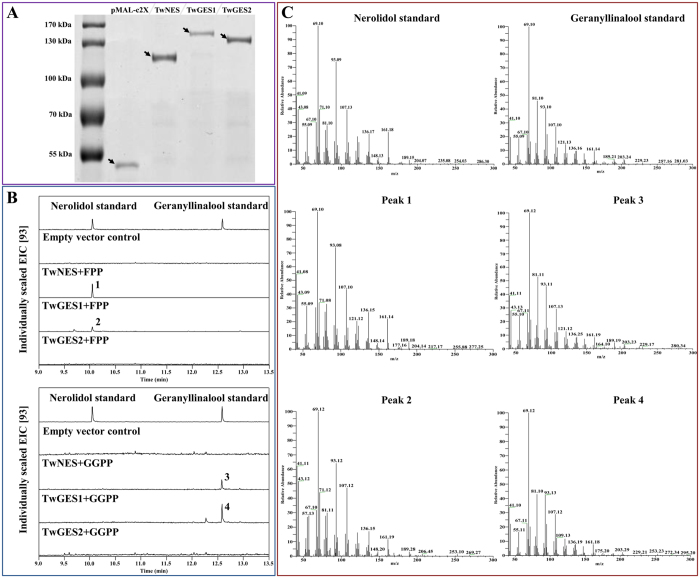
GC-MS analysis of reaction products catalyzed by purified recombinant MBP-tagged proteins with (*E,E*)-FPP and GGPP as substrates. (**A**) SDS-PAGE analysis of recombinant proteins through affinity purification with Amylose Resin. The arrows indicate the purified recombinant proteins; (**B**) Peak 1 was identified as the only product of TwNES with FPP as the substrate, peak 2 was the only product of TwGES1 with FPP as the substrate, peak 3 was the only product of TwNES with GGPP as the substrate, and peak 4 was the only product of TwGES1 with GGPP as the substrate; (**C**). Mass spectrums of peak 1 and 2 were identical to the mass spectrum of the nerolidol standard, and the mass spectrum of peak 3 and 4 were identical to the mass spectrum of the geranyllinalool standard.

The *TwNES* ORF, *TwGES1* ORF and *TwGES2* ORF were cloned into the pMAL-c2X vector and expressed in *E. coli* strain Transetta (DE3) individually for characterization the activities of each protein. Firstly, we induced the expression of recombinant proteins in *E. coli* strain Transetta (DE3) with different induction temperature and time (Supplementary Figure 3), and the results showed that induction at 16 °C for 20 h could get a better result. Under these conditions, all the three proteins have been expressed successfully in solubility and the relative bands of each protein were identified based on the deduced MW and tags (Fig. 3A). The purified proteins were assayed for catalytic activity characterization with respective substrate. The *in vitro* experiments showed both TwNES and TwGES1 were able to catalyze the conversion of (*E,E*)-FPP and GGPP to (*E*)-nerolidol and (*E,E*)-geranyllinalool, respectively. None products generated when TwGES2 was incubated with (*E,E*)-FPP or GGPP (Fig. 3B and C).

**Figure 4:**
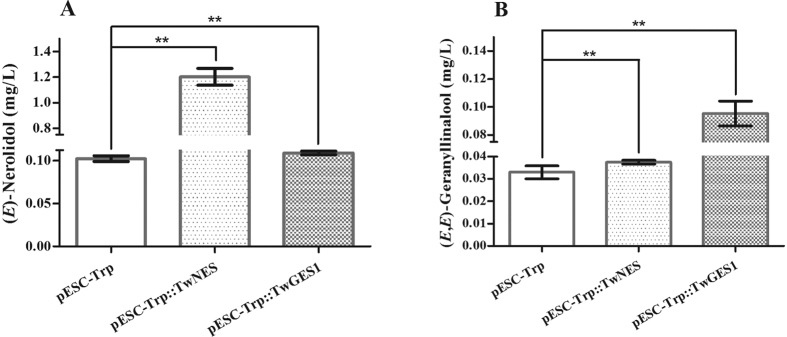
Accumulation of (*E*)-nerolidol and (*E,E*)-geranyllinalool in yeast transformed with TwNES and TwGES1. BY-T20 strain (BY4742, *ΔTrp1, Trp1::HIS3-P*_*PGK1*_*-BTS1/ERG20-T*_*ADH1*_*-P*_*TDH3*_*-SaGGPS-T*_*TPI1*_*-P*_*TEF1*_*-tHMG1-T*_*CYC1*_) containing different recombinant plasmid were cultivated in 100 mL liquid induction medium supplemented with 20 g/L galactose and grown at 30 °C for 3 d^36^. (**A**) Accumulation of (*E*)-nerolidol; (**B**) Accumulation of (*E,E*)-geranyllinalool. pESC-Trp, BY-T20 yeast with pESC-Trp; pESC-Trp::TwNES, BY-T20 with pESC-Trp::TwNES recombinant plasmid; pESC-Trp::TwGES1, BY-T20 with pESC-Trp::TwGES1 recombinant plasmid. Values shown are means ± SD of six replicates (**P < 0.01).

In order to indentify the enzymes more impeccable in eucaryon, we constructed the *TwNES* ORF and *TwGES1* ORF into pESC-Trp vector. The recombinant plastids pESC-Trp::TwNES and pESC-Trp::TwGES1 were transformed into yeast BY-T20, a terpenoids pathway enhanced yeast strain, and the productions after fermentation were detected using GC-MS. In terms of the production of (*E*)-nerolidol, both TwNES and TwGES1 could improve the production significantly. TwNES has a more powerful effect on the accumulation (*E*)-nerolidol, which reached 1.203 mg/L. In terms of the production of (*E,E*)-geranyllinalool, both TwNES and TwGES1 could also improve the production significantly. However, TwGES1 has a more powerful effect on the accumulation (*E,E*)-geranyllinalool, which reached 0.095 mg/L (Fig. 4).

### Enzymatic properties of TwNES and TwGES1

**Figure 5:**
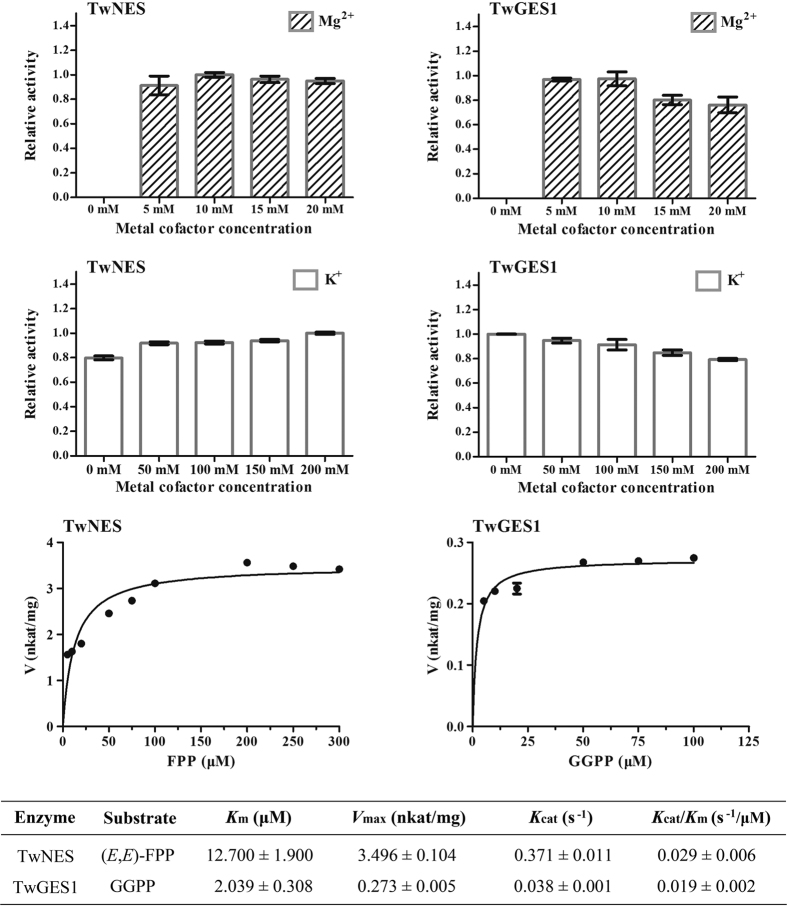
Kinetic analysis of the NES and GES1 purified recombinant proteins. The catalytic activities of the purified enzymes were measured in the presence of the metal ions Mg^2+^ (0, 5, 10, 15 and 20 mM) and K^+^ (0, 50, 100, 150 and 200 mM). Under the optimum concentrations of Mg^2+^ and K^+^ (TwNES, 20 mM MgCl_2_ and 200 mM KCl; TwGES1, 20 mM MgCl_2_), the basic kinetic properties of TwNES and TwGES1 for the substrates (*E,E*)-FPP and GGPP were examined. All assays were incubated for 10 min at 30 °C. *K*_m_ (Michaelis-Menten constant), *V*_max_ (maximal velocity), *K*_cat_ (turnover number) and *K*_cat_/*K*_m_ values of the were calculated by using the GraphPad Prism software. Values shown are means ± SD of three replicates.

The catalytic activities of the purified enzymes were measured in the presence of the metal ions Mg^2+^ and K^+^ at different concentrations. The means and standard errors of each enzyme activity under specific metal ions concentrations are shown in Fig. 5. Determination of the enzymes activities in presence of different concentrations of Mg^2+^ showed that both TwNES and TwGES1 reached the highest activity at 10 mM Mg^2+^, which are similar to those reported enzymes^16^. We further detected the activities in presence of different concentrations of K^+^ with 10 mM Mg^2+^, and TwNES and TwGES1 showed opposite responses to the concentrations of K^+^. TwNES presents the highest activity as the concentration of K^+^ arise (200 mM) while K^+^ inhibits the activity of TwGES1.

Kinetic properties of TwNES and TwGES1 for the substrates (*E,E*)-FPP and GGPP respectively, were examined under the optimum concentrations of Mg^2+^ and K^+^. Kinetic profiles of TwNES for (*E,E*)-FPP were 12.700 μM (*K*_m_), 3.496 nkat/mg (*V*_max_), 0.029 s^−1^/μM (*K*_cat_/*K*_m_) in the presence of 10 mM Mg^2+^ and 200 mM K^+^, and TwGES1 for GGPP were 2.039 μM (*K*_m_), 0.273 nkat/mg (*V*_max_), 0.019 s^−1^/μM (*K*_cat_/*K*_m_) in the presence of 10 mM Mg^2+^ (Fig. 5).

### Expression analysis of *TwNES, TwGES1* and *TwGES2*

**Figure 6:**
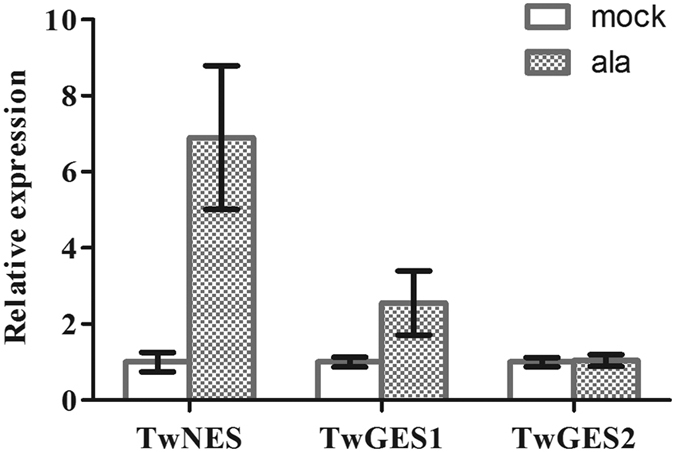
Relative expression of *TwNES, TwGES1* and *TwGES2* in suspension cells treated with ethanol and alamethicin. mock. Control suspension cells were treated with 0.1% ethanol only; ala. Suspension cells treated with alamethicin solution (100 ng/mL, 0.1% ethanol). Means ± SD of triplicate assays are shown.

Previous researches about the expression analyses of *NES* and *GES* mainly concentrated on the plant tissues (e.g. flower and leaf)^7^,^15^,^16^, but no tissue cultures (e.g. suspension cell and hairy root). Relative gene expression analysis was carried out to investigate the expression levels of *TwNES, TwGES1* and *TwGES2* in *T. wilfordii* suspension cells in response to the fungal peptide alamethicin. Interestingly, the transcript levels of *TwNES* and *TwGES1* are increased by 5.9-fold and 1.6-fold respectively in suspension cells treated with alamethicin (ala), compared with control treatment (mock), while *TwGES2* transcript level does not change when treated with alamethicin (Fig. 6).

## Discussion

We have identified three enzymes (TwNES, TwGES1 and TwGES2), involved in the biosynthesis of the C_15_ and C_20_ tertiary alcohols in *T. wilfordii*. Both of TwNES and TwGES1 could catalyze the formation of (*E*)-nerolidol and (*E,E*)-geranyllinalool, whose functions are similar to other NESs and GESs characterized in a number of plant species including *Cinnamomum tenuipilum, Arabidopsis thaliana, strawberry, Vitis vinifera* and *Clarkia breweri*^15^,^16^,^26^,^27^,^28^,^29^, and again demonstrates an inherent capacity for TPS enzymes to evolve different product and substrate specificities^15^. In addition, the activities of enzymes responds to different concentration of metal ions were detected. The optimum Mg^2+^ concentration for TwNES and TwGES1 is 10 mM, which is consistent with previous report about GES and NES^30^. Yang *et al*. reported that additional K^+^ up to 100 mM had no effect on the enzyme activity^26^. However, the metal ion K^+^ used in this paper has apparent influences on the enzyme activities, indicating that TwNES and TwGES1 in *T. wilfordii* are sensitive to K^+^.

TwGES2 lacking a TPS metal binding domain is the firstly reported geranyllinalool synthase among the reported GES from other species (Supplementary Figure 2)^4^,^16^,^25^,^28^,^31^,^32^. The *in vitro* experiments confirm that TwGES2 could not convert (*E,E*)-FPP or GGPP to any products. *TwGES2* expression level does not change in *T. wilfordii* suspension cells when treated with alamethicin, suggested that TwGES2 would not utilize (*E,E*)-FPP or GGPP to form signal products to respond to the exogenous stimuli, further molecularly excluding the involvement of TwGES2 in competing (*E,E*)-FPP or GGPP. In here, we surmised TwGES2 might be a mis-spliced version of TwGES1, or TwGES2 might be a backup enzyme which would mutate into a functional geranyllinalool synthase when TwNES and TwGES1 did not work.

The two identified enzymes TwNES and TwGES1 are most likely to be utilized as gene regulatory elements for further regulating the biosynthesis of the bioactive compounds (e.g. triptolide and celastrol) in *T. wilfordii*. Based on the fact that *TwNES* and *TwGES1* transcript levels are upregulated simultaneously in suspension cells treated with alamethicin, we suppose TwNES and TwGES1 are likely to utilize GGPP and (*E,E*)-FPP to generate tertiary alcohols as precursor of plant volatiles, which play important roles in the ecological interactions between *T. wilfordii* and other organisms, and further experimental evidences are needed for this hypothesis.

## Methods

### Plant materials, substrates and reagents

The *T. wilfordii* suspension cells were cultured in Murashige and Skoog (MS) medium containing 30 g/L sucrose with 0.1 mg/L kinetin (KT), 0.5 mg/L indole-3-butytric acid (IBA) and 0.5 mg/L 2,4-dichlorophenoxyacetic acid (2,4-D), and maintained at 25 ± 1 °C with 120 rpm shaking in the dark as described previously^3^. Farnesyl diphosphate ((*E,E*)-FPP), geranylgeranyl diphosphate (GGPP), (*E*)-nerolidol, (*E,E*)-geranyllinalool standards were purchased from Sigma-Aldrich Co. T4-DNA ligase and restriction enzymes were from New England Biolabs. The *E. coli* strains Trans5α and Transetta (DE3), and pEASY-Blunt Simple Cloning Kit were obtained from TransGen Biotech Co. Ltd. All reagents were purchased from Fisher, unless otherwise noted. Primers were synthesized by Shanghai Sangon Co., and automated DNA sequencing was conducted at Majorbio Co.

### RNA isolation, cDNA synthesis and RACE

Total RNA of suspension cells was extracted using the cetyltrimethylammonium bromide (CTAB) method^33^. The purified RNA was obtained using the RNA Purification Kit to remove genomic DNA (Tiangen Biotech, Beijing, China). The 3′- or 5′-RACE-ready cDNA was reverse transcribed from the purified RNA using the SMARTer^TM^ RACE cDNA Amplification Kit (Clontech Laboratories, Cal., USA). An aliquot (1 μg) of the total RNA was used to synthesize the first strand cDNA according to the PrimeScript 1^st^ Strand cDNA Synthesis Kit manufacturer’s protocol (Takara Bio, Dalian, China).

According to the *T. wilfordii* transcriptome sequencing dataset (not shown), the putative *TwGES* fragment was identified, and the 3′ and 5′ ends of the *TwGES* cDNAs were amplified using the SMARTer^TM^ RACE cDNA Amplification Kit. The 3′ and 5′ gene-specific primers were designed based on this fragment (Supplementary Table 1). All the reactions were performed according to the user’s manual. Primers to amplify the full-length cDNAs for each ORF were designed based on the assembled core fragments of the 3′ and 5′ RACE sequences (Supplementary Table 1). The amplification reactions were performed using PrimeSTAR GXL DNA Polymerase (Takara Bio), according to the manufacturer’s instructions. The PCR products were purified and cloned into the pEASY-Blunt cloning vector, transformed into *E. coli* Trans5α cells, and then cultured in Luria-Bertani (LB) medium (10 g/L Tryptone, 5 g/L yeast extract, 10 g/L NaCl) at 37 °C in the dark. Positive colonies were sequenced. The nerolidol synthase gene (*TwNES*) from *T. wilfordii* was synthesized by Shanghai Sangon Co.

### Recombinant expression and affinity purification

For expression in *E. coli*, the ORFs of NES, GES1 and GES2 were amplified by PCR and subcloned directly into the pMAL-c2X expression vecter (New England Biolabs). Details of the primers are given in Supplementary Table 1. The recombinant plasmids pMAL-c2X::TwNES, pMAL-c2X::TwGES1 and pMAL-c2X::TwGES2 were separately transformed into the *E. coli* strain Transetta(DE3) (TransGen Biotech) for a fusion expression, using the original pMAL-c2X as negative control. Cultures (200 mL) were grown in LB medium containing 100 mg/L ampicillin until optical density of the culture at 600 nm reached 0.6 to 0.8 and then induced with 0.4 mM isopropyl 1-thio-*β*-D-galactopyranoside (Sigma, USA) at 16 °C for 20 h at 200 rpm. The cell pellets were harvested by centrifugation (3000 g, 20 min, 4 °C) and stored at −80 °C until used for affinity purification with Amylose Resin (New England Biolabs).

### Enzymatic assays

Elution buffer containing the purified proteins was exchanged with assay buffer containing 50 mM HEPES, 10 mM MgCl_2_, 100 mM KCl, 5 mM dithiothreitol, and 10% (v/v) glycerol, pH 7.5, using Amicon Ultra-15 centrifugal filter unit with Ultracel-30 membrane (Merck Millipore, Germany), according to the manufacturer’s instructions. Protein concentrations were determined by Bradford method^34^.

To determine the catalytic activity of the recombinant proteins, mixtures of purified protein (20 μg) in 1 mL assay buffer and 25 μM prenyl diphosphate substrates ((*E,E*)-FPP, or GGPP) were overlaid with 400 μL hexane and incubated for 2 h at 30 °C, then thoroughly mixed by vortexing and centrifuged at 8000 g for 2 min, and the supernatant hexane phase was collected. After extraction with hexane (3 × 0.4 mL), the solvent extracts were reduced in volume to 60 μL under a stream of N_2_ before GC-MS analysis as described previously^3^. Linear calibration was performed to calculate the contents of the products (for (*E*)-nerolidol, *R*^2^ = 0.9947 and for (*E,E*)-geranyllinalool, *R*^2^ = 0.9847).

The effect of Mg^2+^ on the enzyme activity was performed at different concentrations of MgCl_2_ (0, 5, 10, 15 and 20 mM, respectively), and the affinity for K^+^ was examined at different concentrations of KCl (0, 50, 100, 150 and 200 mM, respectively). Under the optimum concentrations of Mg^2+^ and K^+^, the basic kinetic properties of TwNES and TwGES1 for the substrates (*E,E*)-FPP and GGPP were examined. All assays were incubated for 10 min at 30 °C. *K*_m_ and *V*_max_ values were calculated by using the GraphPad Prism software. Data were calculated from three independent experiments.

### Yeast expression

The *TwNES* and *TwGES1* ORFs were subcloned into the yeast epitope-tagging vector pESC-Trp under control of the GAL1 inducible promoter (Agilent Technologies, USA) via digestion by the corresponding restriction endonucleases. Expression of TwNES and TwGES1 was performed in the yeast BY-T20 strain (BY4742, *ΔTrp1, Trp1::HIS3-P*_*PGK1*_*-BTS1/ERG20-T*_*ADH1*_*-P*_*TDH3*_*-SaGGPS-T*_*TPI1*_*-P*_*TEF1*_*-tHMG1-T*_*CYC1*_)^35^,^36^,^37^ following the procedure with some modifications, as described previously^38^. The dried samples were dissolved in 60 μL of hexane for GC-MS analysis as described previously^3^.

### Expression analysis of *TwNES, TwGES1* and *TwGES2* performed by RT-PCR

For treatment with the elicitor alamethicin (Sigma-Aldrich), the *T. wilfordii* suspension cells cultured 10 days in Murashige and Skoog (MS) medium as described above were treated with alamethicin solution (100 ng/mL, 0.1% ethanol). Control suspension cells were treated with 0.1% ethanol only (mock). After 30 h treatment at 25 ± 1 °C with 120 rpm shaking, the suspension cells were harvested for real-time gene expression analysis.

Total RNA extraction were performed as mentioned above. First-stand cDNA for real-time quantitative PCR was reverse transcribed from total RNA using the FastQuant RT Kit (Tiangen Biotech). Quantitative real-time PCR was performed using an Applied Biosystems 7300 Real Time PCR System (Applied Biosystems, New York, USA) with the KAPA SYBR^®^ FAST qPCR Kit (KAPA Biosystems, Massachusetts, USA) and gene-specific primers (Supplementary Table 1) to estimate the relative mRNA expression levels. The expression levels of *TwNES, TwGES1* and *TwGES2* were evaluated using the 2^−ΔΔCt^ method^39^ based on *β*-actin as reference gene and triplicate measurements with three biological replicates.

## Additional Information

**How to cite this article**: Su, P. *et al*. Functional characterization of NES and GES responsible for the biosynthesis of (*E*)-nerolidol and (*E,E*)-geranyllinalool in *Tripterygium wilfordii. Sci. Rep.* **7**, 40851; doi: 10.1038/srep40851 (2017).

**Publisher's note:** Springer Nature remains neutral with regard to jurisdictional claims in published maps and institutional affiliations.

## Supplementary Material

Supplementary Information

## Figures and Tables

**Figure 1:**
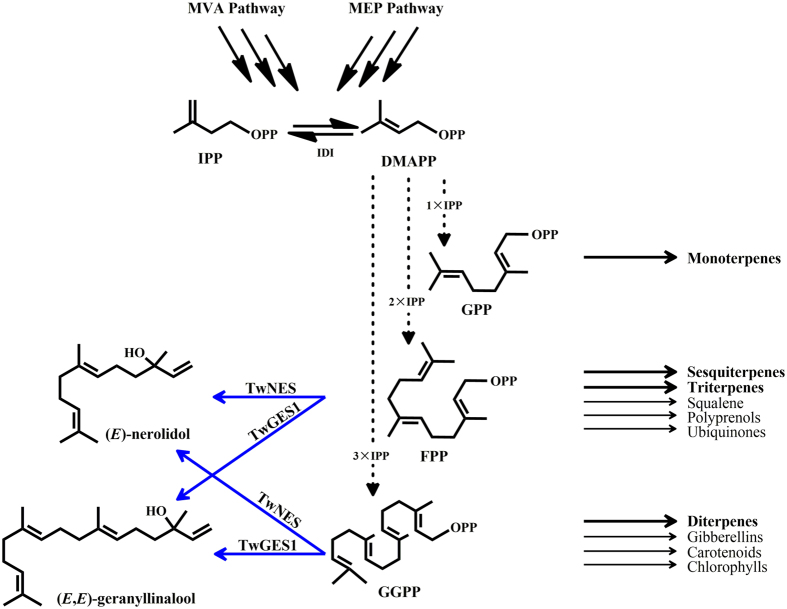
The chemical reactions TwNES and TwGES1 catalyzed in terpenoids biosynthesis pathway. MVA pathway, the cytoplasmic mevalonate pathway; MEP pathway, the plastidic 2-C-methyl-D-erythritol-4-phosphate pathway; IPP, isopentenyl diphosphate; IDI, isopentenyl diphosphate isomerase; DMAPP, dimethylallyl diphosphate; GPP, geranyl diphosphate; FPP farnesyl diphosphate; GGPP, geranylgeranyl diphosphate; NES, nerolidol synthase; GES, geranyllinalool synthase.
