# Supplementary material for: Functional characterization of NES and GES responsible for the biosynthesis of (E)-nerolidol and (E,E)-geranyllinalool in Tripterygium wilfordii
Source: Sci Rep. 2017 Jan 27;7:40851. doi: 10.1038/srep40851 (PMC5269589; doi:10.1038/srep40851)
Supplement: Supplementary Information [file srep40851-s1.pdf]

Functional characterization of NES and GES responsible for the biosynthesis of (*E*)-nerolidol and (*E*,*E*)-geranyllinalool in *Tripterygium wilfordii*

Ping Su[1,2†], Tianyuan Hu[1†], Yujia Liu[1], Yuru Tong[1,2], Hongyu Guan[1,2], Yifeng Zhang[1,2], Jiawei Zhou[1], Luqi Huang[2*], and Wei Gao[1,3*]

[1]School of Traditional Chinese Medicine, Capital Medical University, Beijing, China

[2]State Key Laboratory Breeding Base of Dao-di Herbs, National Resource Center for Chinese Materia Medica, China Academy of Chinese Medical Sciences, Beijing, China

[3]Beijing Key Lab of TCM Collateral Disease Theory Research, Beijing, China

[†]These authors contributed equally to this work

E-mail: huangluqi01@126.com

Tel: +86-10-6401-4411-2955

Fax: +86-10-6401-3996

E-mail: weigao@ccmu.edu.cn

Tel: +86-10-8391-1633

Fax: +86-10-8391-1627

Supplementary Table 1. Primers used in this study

Primer Name	Primer sequence (5`→3`)
**RACE-PCR**	
TwGES-5’	CTGCTTCTGGGTTTTCCTTCAAGTAGAGT
TwGES-3’	TGGCCTAAATGGAGGGTAGATGGGT
**ORF-PCR (pMAL-c2X)**	
TwNES-*BamH*I-F	CGCGGATCCATGGCCTTCTTTGGTTCCTCTCGC
TwNES-*Sal*I-R	CGCGTCGACTTACTTAATCAGGATGGATTTAAGG
TwGES1-*BamH*I-F	CGCGGATCCATGGATTTTTCAGATTCCTCAAT
TwGES1-*Sal*I-R	CGCGTCGACTCAAATGAAACATGGTGAGAATTTTG
TwGES2-*BamH*I-F	CGCGGATCCATGGATTTTTCAGATTCCTCAAT
TwGES2-*Sal*I-R	CGCGTCGACTCAAATGAAACATGGTGAGAATTTTG
**ORF-PCR (pESC-Trp)**	
TwNES-*BamH*I-F	CGCGGATCCGATGGCCTTCTTTGGTTCCTCTC
TwNES-*Apa*I-R	TCGGGGCCCAACTTAATCAGGATGGATTTAAGGAACTC
TwGES1-*BamH*I-F	CGCGGATCCGATGGATTTTTCAGATTCCTCAAT
TwGES1-*Apa*I-R	TCGGGGCCCAAAATGAAACATGGTGAGAATTTTG
**RT-PCR**	
TwNES-F	TGCCGTCCAACGCCTT
TwNES-R	CCATTAGTCCCTTTATGTCTCCA
TwGES1-F	ATTGCTTCACATACTCTACTTCTTCCAG
TwGES1-R	TCTGCTTCTGGGTTTTCCTTCA
TwGES2-F	AAATCTAAAGGAACAAAAGTCAGGG
TwGES2-R	GCATTTGATACACTTTGAGACAAGC
β-actin-F	AGGAACCACCGATCCAGACA
β-actin-R	GGTGCCCTGAGGTCCTGTT

* The underlined bases represent the restriction sites used for cloning. F, forward; R, reverse.

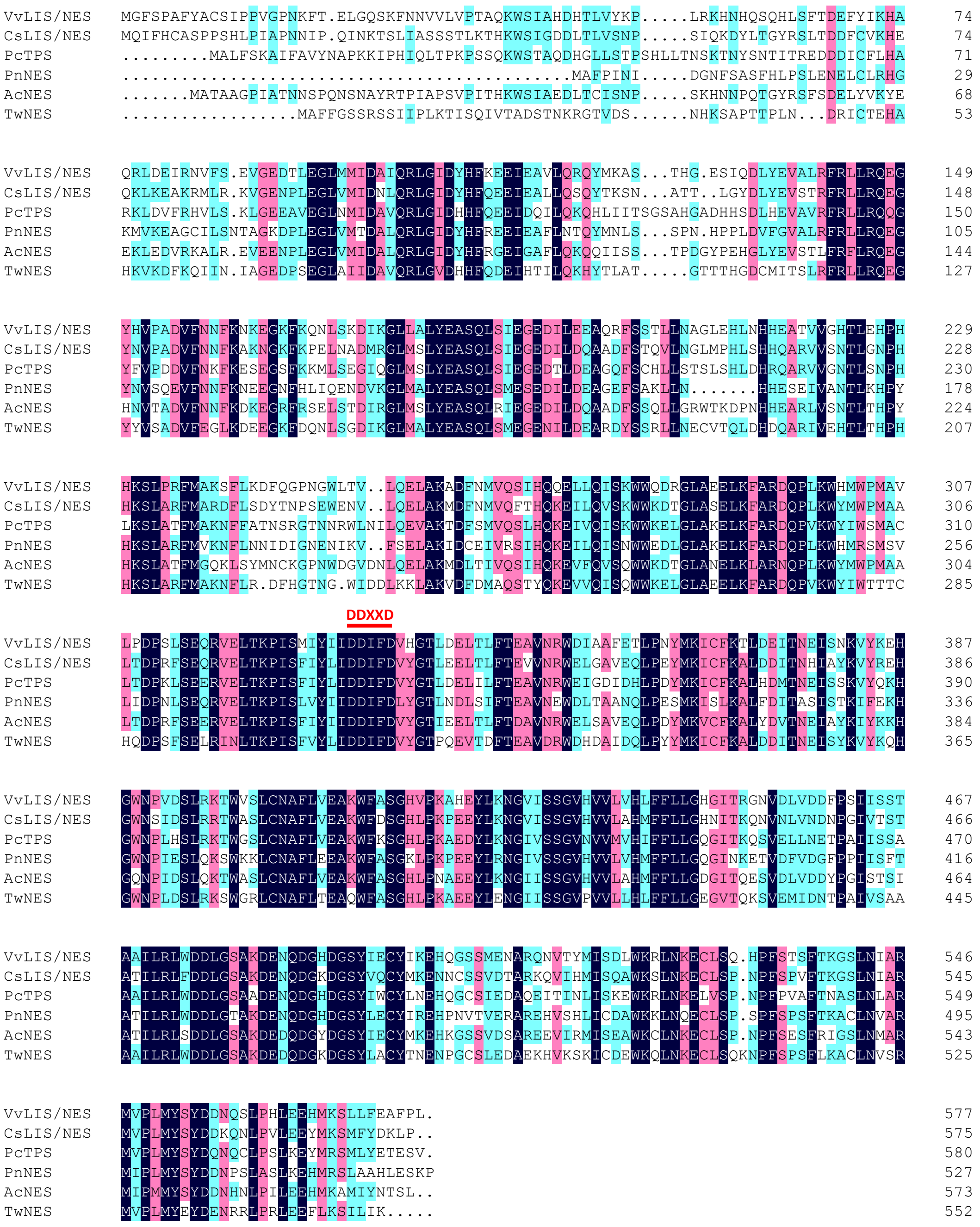

Supplementary Figure 1. Multiple alignments of TwNES. The TwNES sequence was aligned with proteins from *Vitis vinifera* (VvLIS/NES, AEY82696), *Camellia sinensis* (CsLIS/NES, AGX26045), *Prunus cerasoides* var. *campanulata* (PcTPS, AIC76501), *Populus nigra* (PtNES, AHY21667) and *Actinidia chinensis* (AcNES, AER36088). The DDxxD motif is outlined in red.

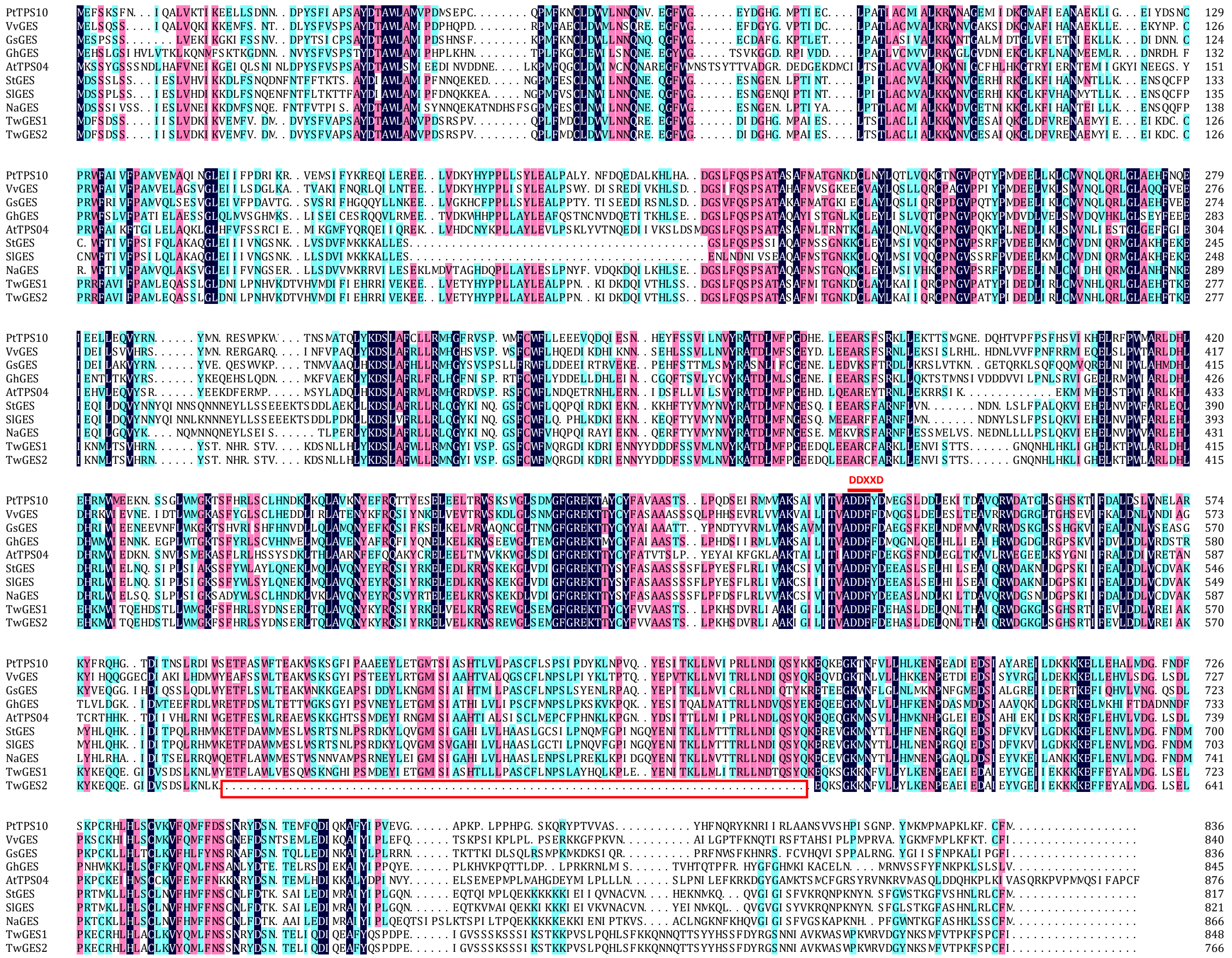

Supplementary Figure 2. Multiple alignments of TwGES1 and TwGES2. The TwGES sequences were aligned with proteins from *Populus trichocarpa* (PtTPS10, AII32474), *Vitis vinifera* (VvGES, NP_001268004), *Glycine soja* (GsGES, KHN46327), *Grindelia hirsutula* (GhGES, AGN70888), *Arabidopsis thaliana* (AtTPS04, NP_564772), *Solanum tuberosum* (StGES, NP_001305587), *Solanum lycopersicum* (SlGES, NP_001289840) and *Nicotiana attenuate* (NaGES, AIL54746). The DDxxD motif and the missing amino acids of TwGES2 are outlined in red.

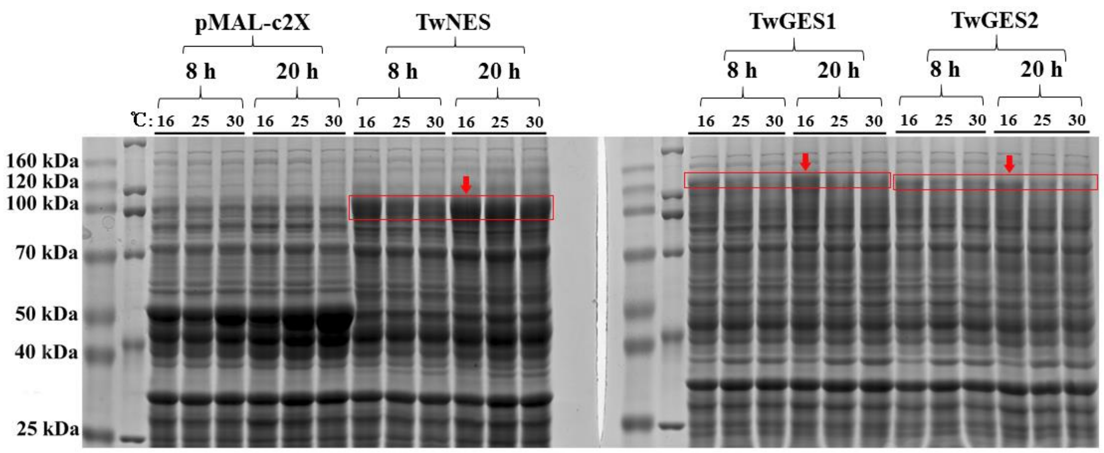

Supplementary Figure 3. SDS-PAGE analysis of recombinant protein expressed in *E. coli* strain Transetta(DE3) with different induction temperature and time.
